# Long-term high-altitude exposure influences task-related representations in visual working memory

**DOI:** 10.3389/fneur.2023.1149623

**Published:** 2023-05-19

**Authors:** Xiaohua Bao, Delong Zhang, Xiaoyan Li, Ming Liu, Hailin Ma

**Affiliations:** ^1^Faculty of Psychology, Tianjin Normal University, Tianjin, China; ^2^Plateau Brain Science Research Center, South China Normal University, Guangzhou, China; ^3^Center for the Study of Applied Psychology, Key Laboratory of Mental Health and Cognitive Science of Guangdong Province, School of Psychology, South China Normal University, Guangzhou, China; ^4^Plateau Brain Science Research Center, Tibet University, Lhasa, China; ^5^Academy of Plateau Science and Sustainability, People's Government of Qinghai Province, Xining, China

**Keywords:** high altitude, hypoxia, working memory, contralateral delay activity (CDA), memory load

## Abstract

**Objective:**

Human working memory is impaired when individuals are exposed to high altitudes, however, whether the capacity of visual working memory is affected remains unclear. This study combined a lateralized change detection task and event-related potentials analysis to explore changes in visual working memory capacity among individuals who emigrated from a low-altitude environment to Tibet (a high-altitude environment).

**Materials and methods:**

Thirty-five college students were recruited from Tibet University as the high-altitude (HA) group, and thirty-six low-altitude (LA) students were enrolled from South China Normal University (sea level) as the LA group. We measured participants' contralateral delay activity (CDA) under different memory loads.

**Results:**

ERP component analysis showed that both the HA and LA groups reached an asymptote at memory load four. However, the contralateral and ipsilateral activity of the HA and LA groups shows different patterns. The results showed a significantly larger contralateral activity for the LA group than for the HA group at memory load one (*p* = 0.04, Cohen's *d* = 0.52) and load three (*p* = 0.02, Cohen's *d* = 0.61). Additionally, we found marginally larger contralateral activity at memory load four for the LA group (*p* = 0.06, Cohen's d = 0.47), but not at memory load two (*p* = 0.10) or load five (*p* = 0.12). No significant differences were observed for ipsilateral activity. In addition, we observed that the HA group performed larger ipsilateral activity than contralateral activity under each memory load, compared with the LA group.

**Conclusion:**

These findings demonstrated that the attentional resource of long-term HA exposure is more captured by task-irrelevant information, potentially due to impaired inhibitory control, which makes it difficult for them to exclude the interference of task-irrelevant information.

## 1. Introduction

The human brain is known to be sensitive to environmental changes and stressors (1, 2). A growing body of research has demonstrated that exposure to high-altitude (HA) environments can have an impact on cognitive functions (3) such as attention (4) and working memory (5, 6).

Working memory is a crucial system for higher-level cognitive processes, yet it has a limited capacity to process diverse representations of multiple objects (7–9). Classical theories propose that working memory is composed of multiple subsystems, including a phonological loop for processing verbal stimuli, a visuospatial sketchpad for handling spatial information, and a central executive system consisting of three cognitive processes: shifting, updating, and inhibition (7–9). Many studies have indicated that the phonological loop is responsible for verbal working memory, while the visuospatial sketchpad has been associated with spatial working memory (6). Previous research on working memory has primarily focused on its executive function. For example, one study demonstrated that prolonged exposure to HA led to decreased response accuracy and increased reaction time in verbal working memory tasks (5).

Moreover, the impact of HA exposure on the updating function may be attributed to the impairment of both the matching process and the storage process in both verbal and spatial working memory (5). Current research on verbal and spatial working memory has focused on the influence of executive function on these two systems, which is because tasks with lower memory loads can be completed by a single component of the working memory system, either verbal or spatial working memory (10). However, as the memory load increases, the information processing within working memory requires the coordinated operation of multiple subsystems, including the phonological loop, the visuospatial sketchpad, and the central executive system (10). Thus, studies exploring the relationship between hypoxia and working memory should not overlook the significance of working memory capacity.

The size of working memory capacity is critical to completing working memory tasks. It has been demonstrated that working memory can only retain three to four objects simultaneously at any given time. Furthermore, working memory capacity has a strong correlation with inhibitory abilities in cognitive control tasks, such as the flanker task (11, 12), oddball task (13), and anti-saccade tasks (14). This evidence supports the idea that impaired inhibition control may be related to reduced working memory capacity. Inhibitory control is the ability to filter out task-irrelevant information. To some extent, the size of working memory capacity depends on this ability, as successfully completing the encoding and retrieval stages of working memory always requires the suppression of irrelevant information and the ability to prioritize items that need to be remembered (14, 15). In previous studies, it has been observed that prolonged exposure to HA can result in a decrease in working memory capacity, as indicated by a decline in inhibition control (11, 16, 17).

Lateralized change detection tasks and event-related potentials are widely used to investigate the neural activity differences associated with visual working memory (VWM) capacity in individuals. The amplitude of the contralateral delay activity (CDA) reflects an individual's VWM capacity, which is moderated by the amount of capacity in visual-spatial working memory. CDA is a sustained negative voltage derived from posterior electrodes, and a different form of wave is generated by subtracting the ipsilateral activity from the contralateral activity, and the amplitude of the resulting waveform represents the strength of the CDA (15). The contralateral activity represents the neural activity observed on the hemisphere contralateral to the to-be-remembered side, while the ipsilateral activity represents the neural activity on the hemisphere ipsilateral to the to-be-ignored side (18–20). The amplitude of the CDA is indicative of VWM capacity and increases proportionally with an increase in the number of items to be remembered, reaching an asymptote at the maximum storage threshold of working memory capacity (18, 21). In addition, previous studies have commonly assumed that the contralateral and ipsilateral amplitudes of the CDA correspond to the storage of task-relevant items and the processing of task-irrelevant items, respectively (20, 22). Thus, utilizing these markers could help us determine the effects of long-term HA exposure on working memory and provide new evidence regarding the impairment of inhibition caused by long-term HA exposure.

In studies related to VWM, the *K*-score has been proposed as an indicator of individuals' performance effectiveness. As it is highly correlated with the ability to filter our task-irrelevant information, any impairment in the ability to filter our task-irrelevant information in individuals exposed to HA may be reflected in their *K*-score assessments. To examine the impact of hypoxia on VWM, we employed the lateralized change detection task and the *K*-score to evaluate individuals who had resided in a HA environment for 3 years after emigrating from a lower altitude region. A previous study found that students who had lived in HA areas for 2 years would develop an adaptive mechanism to cope with hypoxia. However, this adaptation is remarkably similar to the compensatory mechanism of Tibetans through natural selection (23). The adaptation to HA by immigrants allows us to understand the long-term effects of HA exposure on VWM capacity and intervene in such adaptive changes. The pieces of evidence from electrophysiological studies suggested that prolonged exposure to HA mainly impairs the late processing stage of cognition due to insufficient attention allocation (24–26). Contralateral and ipsilateral activity can effectively reflect whether HA continues to pay attention to task-related information and whether task-irrelevant information affects HA as memory load increases. Thus, we hypothesized that individuals in the HA group would reach an asymptote in the amplitude of their CDA with a lower working memory load than those in the low altitude (LA) group. Additionally, due to impaired inhibition control, the HA group will exhibit greater ipsilateral activity than contralateral activity compared to the LA group.

## 2. Materials and methods

### 2.1. Participants

Previous studies have found that students who immigrated to HA areas for 2 years showed similar brain mechanisms to Tibetans for adapting to a hostile HA environment. However, immigrant students have lower SpO_2_ levels than Tibetans (17, 23), suggesting HA immigrants were suffering from hypoxia.

The present study employed a mixed experimental design of 2 (group: HA vs. LA) × 5 (memory load: 1–5), with the group as a between-subjects factor and memory load as a within-subjects factor. According to the analysis conducted with G^*^power 3.1 (http://www.gpower.hhu.de/), a sample size of 42 participants would yield 95% power to detect a medium effect size (*d* = 0.25) at a significance level of 0.01. A total of 35 college students were recruited from Tibet University as the HA group. They had lived in low-altitude areas (below 1,500 m) from birth until adulthood and had immigrated to a high-altitude area to attend college (Lhasa, 3,650 m), where they had resided for the past 3 years. The LA group consisted of 34 college students from South China Normal University (sea level). The participants were junior and senior students (aged 19–22 years) and were counterbalanced based on their demographic information (Table 1). All the participants in the present study were of Han ethnic background. All participants had normal or corrected-to-normal vision and had no history of mental illness or regular medication use. Six participants were excluded due to low accuracy and artifacts in their electroencephalogram (EEG). Data from 32 participants in the HA group and 31 participants in the LA group were enrolled in the main analysis. The experiment was conducted in compliance with the guidelines established by the Declaration of Helsinki. Our study design received approval from the ethics committee of South China Normal University. All participants provided informed consent before the experiment and were informed that they would be compensated upon the completion of the study.

**Table 1:** Gender, exam score, and intelligence scores (Raven progress matrices) of each group.

	**High altitude**	**Low altitude**	***P-*value**
Gender (M/F)	15/17	16/15	0.4
Exam score	500.16 ± 55.1	513.5 ± 111.2	0.6
Intelligence	38.8 ± 4.9	41.5 ± 6.5	0.07

M, male; F, female; exam score refers to the scores of the college entrance examination.

### 2.2. Materials and procedure

**Figure 1:**
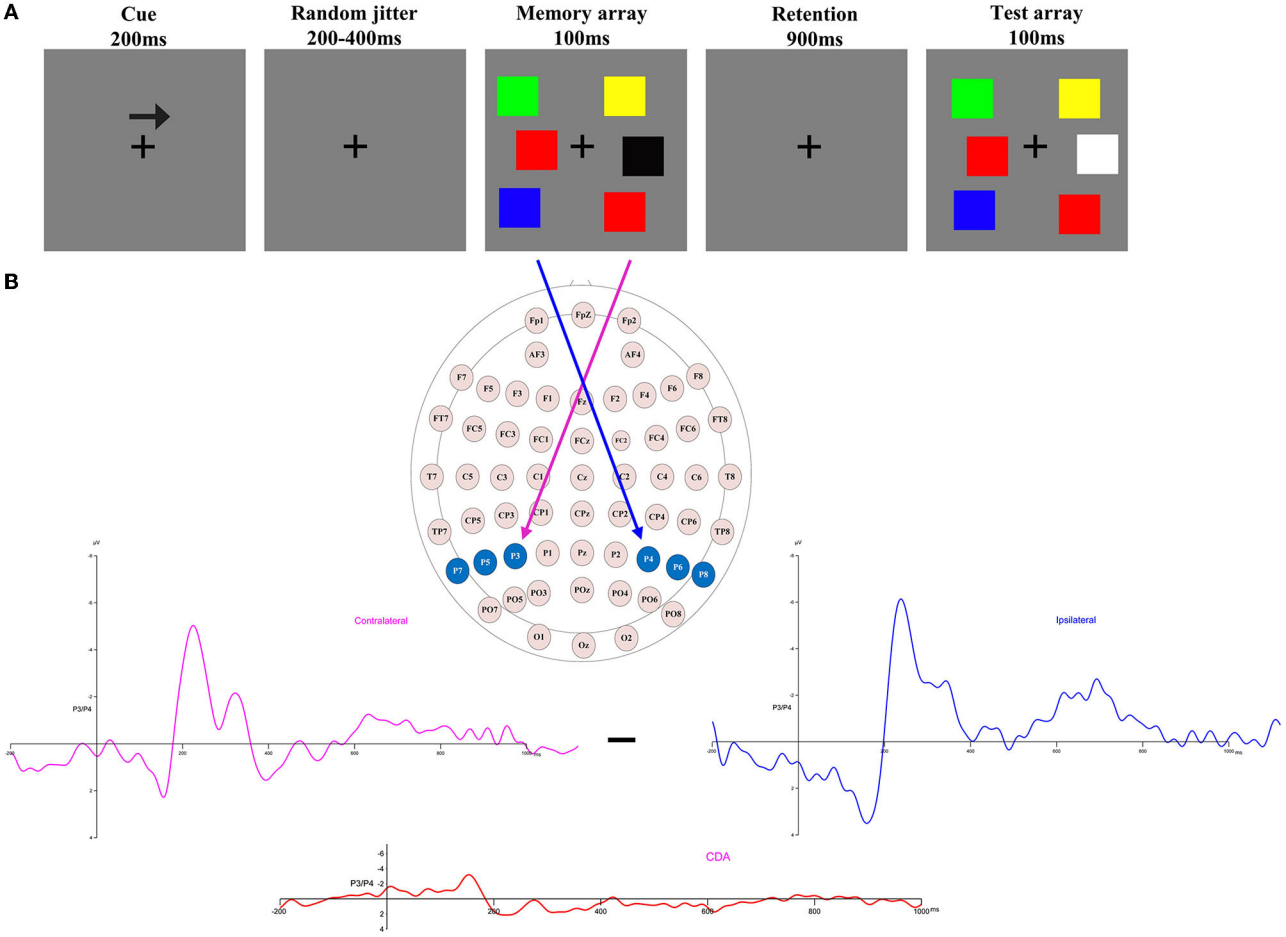
**(A)** Trial structure of the change detection task. A change trial with a memory load of three in which the colors of the right items are to be remembered (as indicated by the arrow cue). **(B)** Electrode sites were used in the ERP component analysis and the calculation of contralateral, ipsilateral, and CDA waveforms when the items to be remembered were presented to the right of fixation [adapted from (27)].

The lateralized change detection task involved a simple bilateral target display (1–5 colored squares) where both sides were identical (Figure 1A) (18, 20, 28). The memory array and test array were presented within 4° × 7.6° rectangular regions, which displayed 2.8° of the array to two sides of a central fixation cross; the background was gray. The size of each square in the memory array and test array was 0.68° × 0.68°, and they were randomly colored using seven highly perceptually distinguishable colors: green (RGB values: 0, 255, 0), red (RGB values: 255, 0, 0), blue (RGB values: 0, 0, 255), purple (RGB values: 160, 32, 240), black (RGB values: 0, 0, 0), yellow (RGB values: 255, 255, 0), and white (RGB values: 255, 255, 255). Each color was presented once within a rectangular region, the positions of the squares varied randomly, and the constrained distance between squares within a hemifield was no < 2°.

Each trial of the lateralized change detection task began with a 200-ms arrow cue, instructing participants to remember the lateralized colors in the direction of the arrow in the memory array. This was followed by a variable interval of 200–400 ms to allow the participants to prepare for the task. Next, a memory array was presented for 100 ms, after which participants were given a retention (delay) period of 900 ms. Finally, the test array was presented for a maximum duration of 5,000 ms, and participants were required to press different keys based on the colors of the to-be-remembered items in the memory array to match them with the colors in the test array. Only half of the trials were consistent. The instructions highlighted guaranteed accuracy rather than speed. The duration of the inter-trial interval was 2,000 ms.

The memory load was randomly presented in each of the blocks. There were 12 blocks, each containing 80 trials, and each memory load consisted of 192 trials. Before the formal experiment, participants completed a practice block consisting of 20 trials (each memory load containing four trials). In the EEG recording process, the participants were instructed to focus on a fixed point (a cross at the center of the array) and to minimize blinking during each trial as much as possible.

### 2.3. EEG recording and processing

The EEG data were recorded using 64-channel Ag/AgCl electrodes mounted in an elastic cap (Curry7.0) and re-referenced by the average of the left and right mastoids (M1 and M2), with the physical reference being a ground electrode ~2 cm posterior to CZ. The two electrodes that were placed above and below the left eye were used to record vertical electrooculograms (EOGs). The horizontal electrooculogram (HEOG) was monitored using a pair of electrodes placed 10 mm from the right and left orbital rims. The impedance of all inter-electrodes was kept below 5 kΩ, and the EEG signals were filtered using a bandpass filter at 0.1–30 Hz with a digitizing rate of 500 Hz using a SynAmp amplifier. We averaged the left and right mastoid (M1 and M2, respectively) using offline re-referenced EEG data.

Ocular artifacts of the EEG signals were removed in the Curry7.0 by covariance analysis. Trials containing muscle artifacts (exceeding ±100 μV for all electrodes) and eye blinks (vertical EOG exceeding ±75 μV) were removed from further analyses. In the high-altitude group, 8.6% of trials were excluded due to artifacts, while 8.4% of trials in the low-altitude group were excluded. In event-related potential (ERP) analysis, only correct trials were included.

### 2.4. Measures and analyses

In our study, the main behavioral measure was the *K*-score, which represents the ability to store and retrieve working memory. The whole-display probe method was used in our experiment to calculate the *K*-score (18), where *K* = *N* × (HR – FA)/(1 – FA), where *K* represents working memory capacity and *N* represents the total number of the items to be remembered. In the present study, the next trial would only commence when participants pressed a key. HR refers to correct responses when the test array changes, and FA refers to incorrect responses on inconsistent trials. We conducted a 2 × 5 mixed analysis of variance (ANOVA) to analyze the *K*-score, with the group (HA and LA) as the between-subjects factor and memory load (1–5) as the within-subjects factor. Similarly, we used a mixed ANOVA to analyze the mean accuracy rates for the *K*-score.

**Figure 2:**
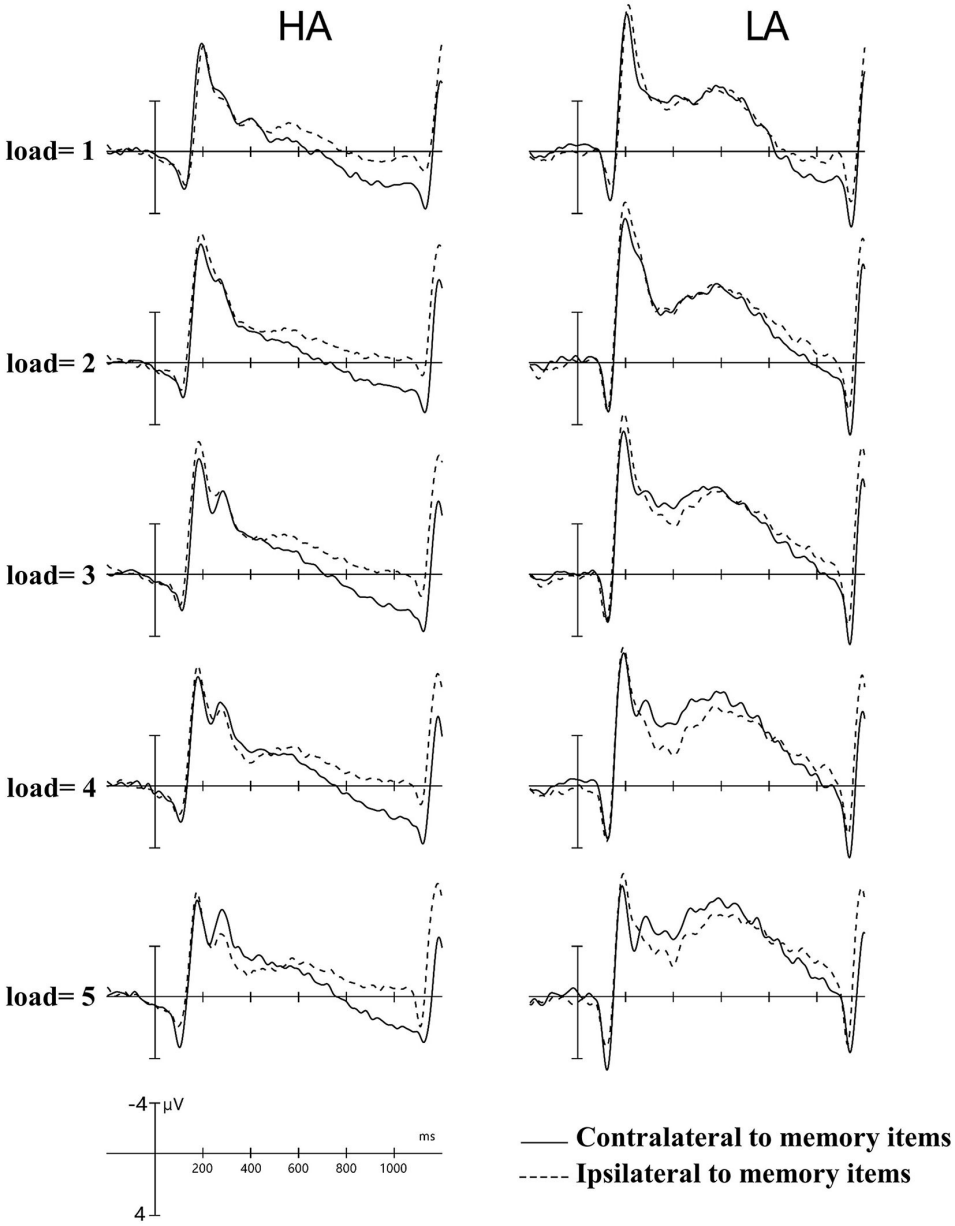
Contralateral and ipsilateral waveforms. Collapsed across posterior electrode sites for each group at each memory load between −200 and −1,200 ms. HA, high altitude; LA, low altitude.

The analytical epoch was 1,400 ms of continuous EEG, starting 200 ms before the pre-memory array and ending 1,200 ms after the memory array onset. Averaged waveforms were calculated for each participant for each memory load (1–5) and CDA. The CDA is obtained by subtracting ipsilateral activity from contralateral activity. The definitions of contralateral and ipsilateral activity are based on the presentation location of the memory items relative to a specific electrode site. The contralateral waveform was calculated by averaging over both to-be-remembered sides. The ipsilateral was computed by averaging over both to-be-neglected sides while factoring in the corresponding channel location. For instance, when computing a neural activity, e.g., P3, which was located to the left on the scalp, the opposite side of the arrow cue, that is, P4, was defined as contralateral. In contrast, the side that corresponds to the arrow cue, that is, P3, was categorized as ipsilateral. For the analog electrode P4, located to the right on the scalp, the opposite schema applies (Figure 1B). Figure 2 displays the contralateral and ipsilateral waveforms resulting from the average of each paired electrode site located posteriorly across the hemisphere for each group within each memory load. The amplitude of the CDA was calculated based on the hemisphere's activity during the retention period, whether it was ipsilateral or contralateral to the memory items. The CDA component was obtained by averaging the activity of three pairs of posterior electrodes (P3/4, P5/6, and P7/8) within a time window of 300–450 ms after the onset of the memory array (18, 21). **Figure 4** displays the inter-posterior electrode pair amplitudes for each group and memory condition separately. We analyzed the amplitudes of the CDA by entering them into a 2 × 5 mixed ANOVA, with the group (HA and LA) as the between-subjects factor and memory load (1–5) as the within-subjects factor.

## 3. Results

### 3.1. Behavioral results

The data analyses were performed using IBM SPSS Statistics 25 (IBM Corp., released in 2017). A significance level of 0.05 was used for all analyses, and the Greenhouse–Geisser correction was applied to correct for sphericity violations whenever appropriate. A *post-hoc* test of the significant main and interaction effects was applied using Bonferroni adjustments. The partial eta-squared ($\eta_{p}^{2}$) values were calculated and provided to demonstrate effect size in ANOVAs, while Cohen's d was employed to show effect sizes in multiple critical comparisons.

**Figure 3:**
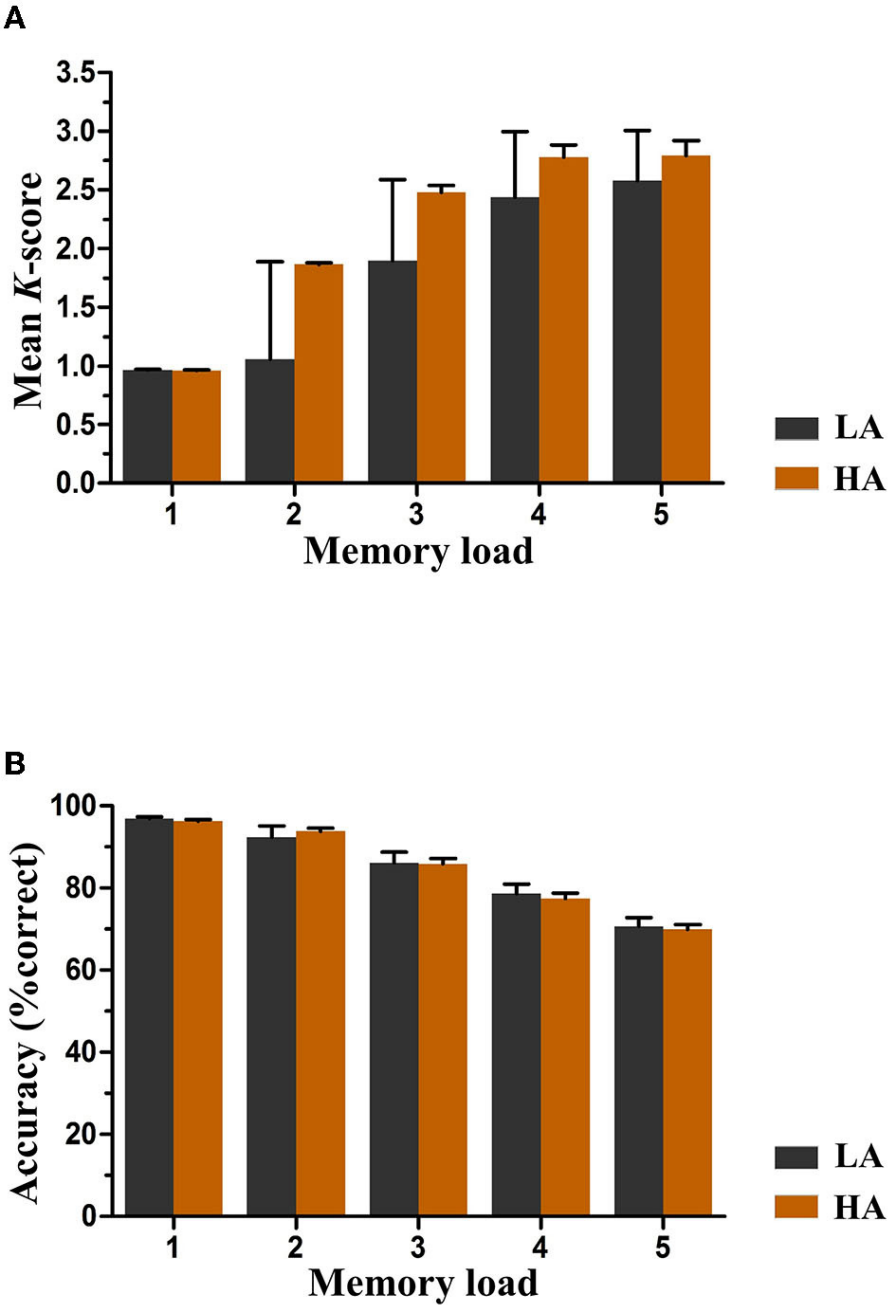
Mean *K*-scores **(A)** and accuracy **(B)** as a function of group and memory load. Error bars represent standard errors of the means. HA, high altitude; LA, low altitude.

Pashler's *K-*scores varied with memory loads and were a function of group and memory load, as depicted in Figure 3A. The analysis of *K*-scores revealed no significant main effect of group, *F*_(1, 61)_ = 0.58, *p* = 0.45, and the interaction between group and memory load for *K* scores was also not significant, *F*_(4, 244)_ = 0.96, *p* = 0.34. However, we observed a significant main effect of memory load *F*_(4, 244)_ = 18.57, *p* < 0.001, $\eta_{p}^{2}$ = 0.23, indicating an increase in *K*-scores with an increase in the number of items to be remembered. Further *post*-*hoc* tests showed that the increase in *K*-scores was significant between memory loads two and four (*p* < 0.01), which were approximately equal to load five (*p* = 0.55). Based on the data, it was observed that the maximum *K*-scores for both groups were achieved when the memory load was four. The average capacity for HA immigrants was estimated to be 2.76 items, and for LA individuals, it was estimated to be 2.61.

Figure 3B displays the accuracy rates of both groups for each memory load. A mixed ANOVA was conducted to analyze the mean accuracy, where memory load was the within-subjects factor and group (HA and LA) was the between-subjects factor. The results showed no significant main effect of group or interaction effect between group and memory load [*F*_(1, 61)_ = 0.05, *p* = 0.82, and *F*_(4, 244)_ = 0.74, *p* = 0.47, respectively] for any memory load, indicating equivalent performance levels between both groups. Nevertheless, a significant main effect of memory load was observed, suggesting a significant increase in accuracy rates as the number of items to be remembered increased, *F*_(4, 244)_ = 240.45, *p* < 0.001, $\eta_{p}^{2}$ = 0.80.

### 3.2. CDA results

**Figure 4:**
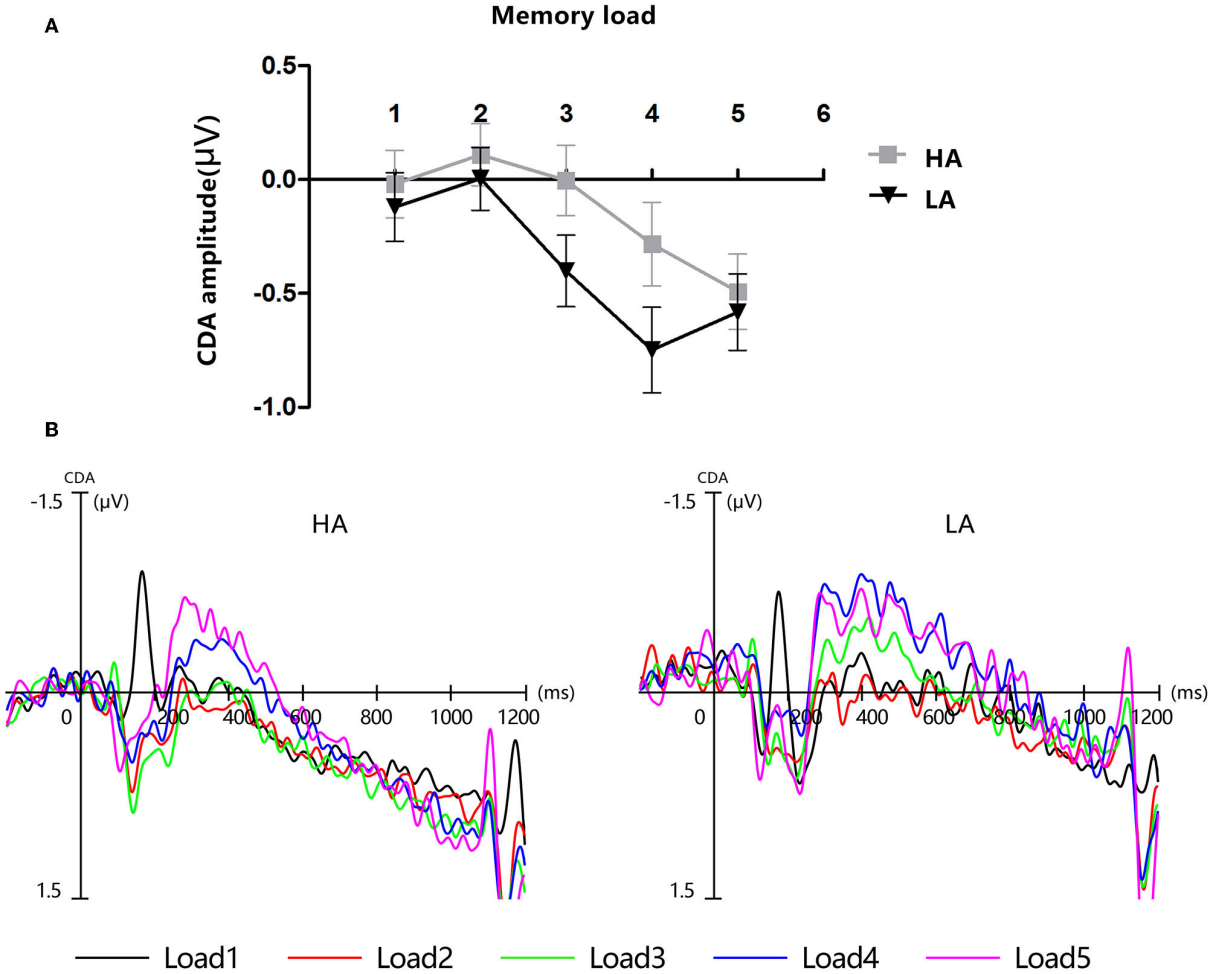
Contralateral delay activity (CDA). HA, high altitude; LA, low altitude. **(A)** Mean amplitude of CDA between 300 and 450 ms after memory array onset. Error bars represent standard errors of the means. **(B)** Grand-average event-related potential waveforms time-locked to memory array onset show the contralateral delay activity (CDA) difference waves for each group at each memory load.

**Figure 5:**
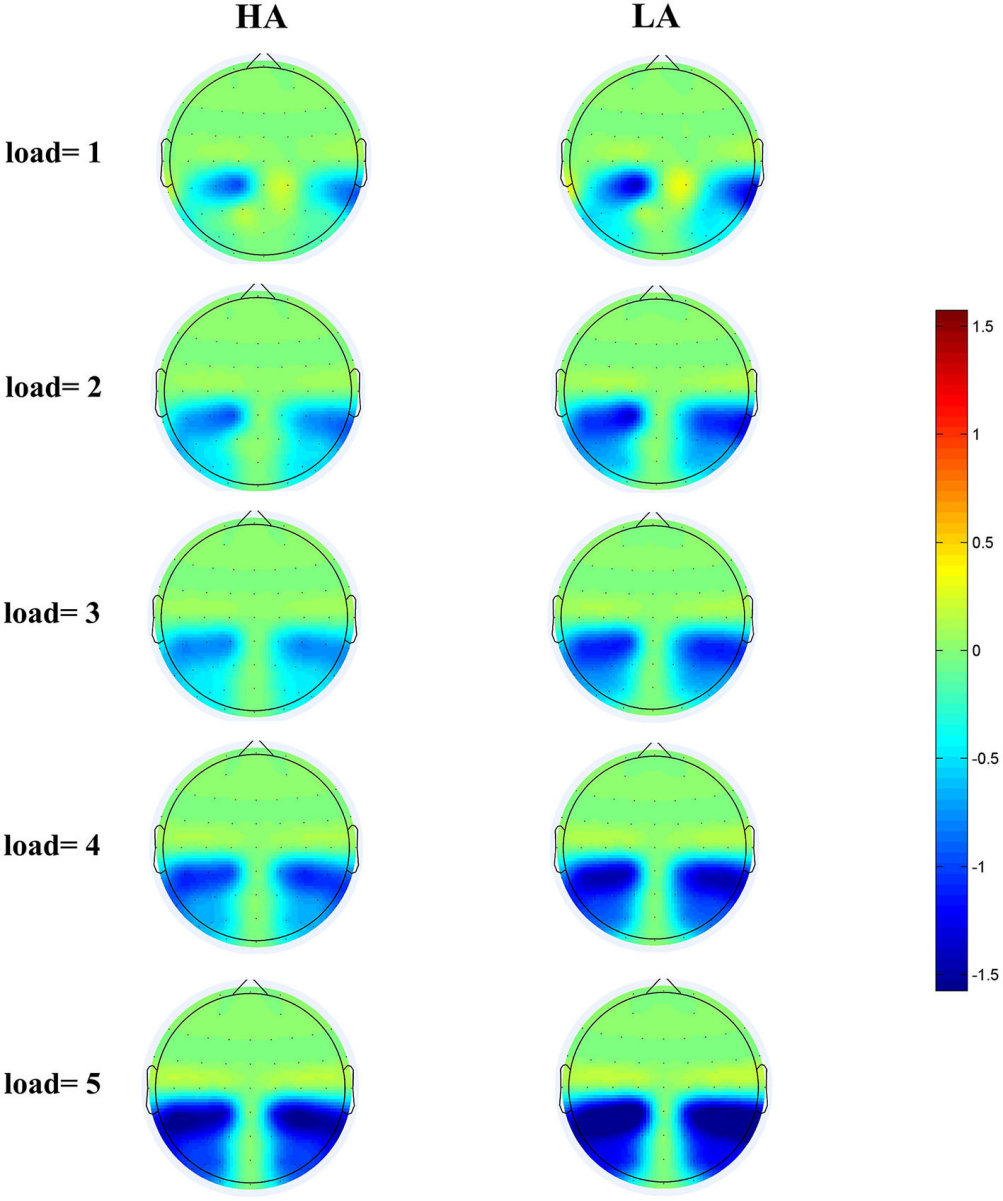
Topographical maps of the CDA at posterior electrode sites during the 300–450 ms time window for HA **(left)** and for LA **(right)** at five memory loads.

Figure 4 displays the grand-average amplitude of the CDA for both groups under each memory load. Figure 5 displays the topographical maps of the CDA at posterior electrode sites, corresponding to the time window of the load-dependent effect of CDA amplitude. The repeated measures ANOVA revealed a marginally significant interaction effect between memory load and group, *F*_(4, 244)_ = 2.29, *p* = 0.08, $\eta_{p}^{2}$ = 0.04. The main effect of memory load was significant, *F*_(4, 244)_ = 19.05, *p* < 0.001, $\eta_{p}^{2}$ = 0.02, and the main group effect was not significant, *F*_(1, 61)_ = 1.37, *p* = 0.25. To examine the maximum asymptote of the CDA in both groups, a *post-hoc* test was conducted for each group. The results showed that, in the HA group, there was a significant increase in CDA amplitudes from load three to load four (*p* = 0.02, Cohen's *d* = 0.28) but no significant change from load one to load two (*p* = 0.23), load two to load three (*p* = 0.21), or load four to load five (*p* = 0.09). Similarly, in the LA group, there was a significant increase in the CDA amplitudes from load two to load three (*p* < 0.001, Cohen's *d* = 0.52) and from load three to load four (*p* = 0.004, Cohen's *d* = 0.38), but no difference in the CDA amplitude from load one to load two (*p* = 0.26) or from load two to load five (*p* = 0.17). These findings suggest that the CDA amplitudes of both groups reached their maximum asymptotes at a memory load of four.

**Figure 6:**
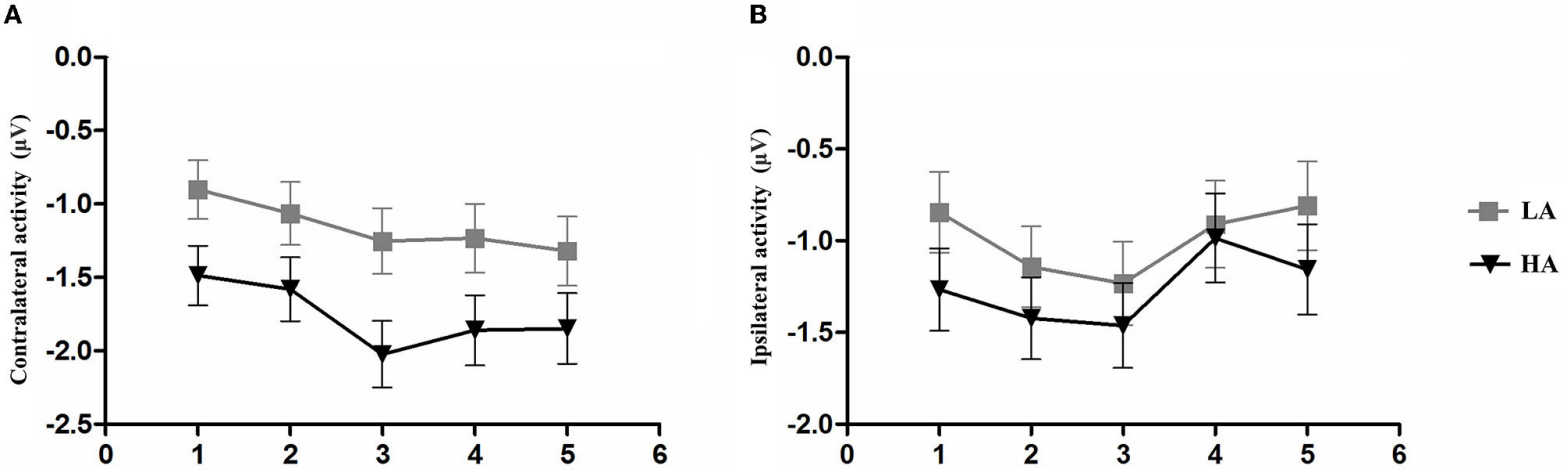
Mean amplitude of contralateral activity and mean amplitude. The epoch is 300–450 ms after memory array onset as a function of group and memory load. **(A)** Mean amplitude of contralateral activity. **(B)** Mean amplitude of ipsilateral activity. Error bars represent standard errors of the means.

To further clarify whether there were differences between the two groups in contralateral and ipsilateral activity within the time window of the load-dependent effect of CDA amplitude between 300 and 450 ms, we computed the lateralized ERPs of contralateral and ipsilateral activity of the cued hemifield. Figure 6 shows the mean amplitudes of contralateral and ipsilateral activity. The analysis of a mixed ANOVA of within-subject factors, memory load (1–5), hemisphere (contralateral/ipsilateral), and the between-subject factor group (HA and LA) was conducted. There was no significant interaction effect with group × hemisphere × memory load *F*_(4, 244)_ = 2.29, *p* = 0.08, but we found a significant interaction between memory load and group, *F*_(4, 244)_ = 19.05, *p* < 0.001, $\eta_{p}^{2}\;=$ 0.24.

A *post-hoc t*-test was conducted to examine the contralateral and ipsilateral activity of the HA and LA groups. The results showed a significantly larger contralateral activity for the LA group than for the HA group at memory load one (*p* = 0.04, Cohen's *d* = 0.52) and load three (*p* = 0.02, Cohen's *d* = 0.61). Additionally, we found marginally larger contralateral activity at memory load four for the LA group (*p* = 0.06, Cohen's *d* = 0.47), but not at memory load two (*p* = 0.10) or load five (*p* = 0.12). No significant differences were observed for ipsilateral activity.

Figure 2 shows the amplitude of ipsilateral waveforms larger than that of the ipsilateral waveforms of LA within the time window of 600–1,000 ms. Thus, we computed the lateralized ERPs of contralateral and ipsilateral activity of the cued hemifields of both groups. A mixed ANOVA was conducted to analyze the mean amplitudes of contralateral and ipsilateral activity with memory load and hemisphere (contralateral/ipsilateral) as within-subject factors and group (HA and LA) as between-subject factors. The results showed there was no significant interaction effect of group × hemisphere × memory load, *F*_(4, 244)_ = 1.27, *p* = 0.29, but we observed a significant interaction effect between hemisphere × memory load, *F*_(4, 244)_ = 6.13, *p* < 0.001, $\eta_{p}^{2}\;=$ 0.09. Subsequent *post-hoc* analysis showed that the amplitude of ipsilateral waveforms was significantly larger than the amplitude of contralateral, ipsilateral waveforms for HA at each memory load (Table 2), whereas there was no significant difference between the amplitude of ipsilateral and contralateral waveforms when memory loads were one, two, four, and five, but the amplitude of ipsilateral waveforms was significantly larger than the contralateral waveform at memory load three for LA. These results indicated that HA immigrants processed task-irrelevant information deeper than task-relevant information compared to LA individuals.

**Table 2:** ERPs amplitudes for the HA and LA groups across memory loads during 300–450 ms (M ± SD).

**Memory load**	**CDA**		**Contralateral activity**		**Ipsilateral activity**	
	**HA**	**LA**	**HA**	**LA**	**HA**	**LA**
One	−0.019 (0.80)	−0.12 (0.88)	0.9 (0.2)	1.5 (0.2)	−0.9 (0.2)	−1.4 (0.2)
Two	0.109 (0.81)	0.004 (0.73)	1.1 (0.2)	1.6 (0.2)	−1.2 (0.2)	−1.6 (0.2)
Three	−0.003 (0.91)	−0.401 (0.84)	1.3 (0.2)	−2.0 (0.2)	−1.3 (0.2)	−1.6 (0.2)
Four	−0.283 (1.08)	−0.748 (0.998)	1.2 (0.2)	−1.9 (0.2)	−0.9 (0.2)	−1.1 (0.2)
Five	−0.492 (0.86)	−0.582 (1.001)	1.3 (0.2)	−1.8 (0.2)	−0.8 (0.2)	−1.3 (0.3)

In Figure 2, it can be observed that, within the time window of 300–1,200 ms, we conducted a repeated ANOVA to examine whether the amplitude of ipsilateral and those contralateral waveforms was larger than that of HA. The results showed there was a significant interaction effect of group × hemisphere × memory load, *F*_(4, 244)_ = 2.66, *p* = 0.04, $\eta_{p}^{2}\;=$ 0.04. The *post-hoc* analysis showed that the amplitudes of ipsilateral and contralateral waveforms in LA were significantly larger than those in HA (Table 3). These results suggest that LA individuals may process task-relevant and task-irrelevant information deeper than HA immigrants.

**Table 3:** Contralateral and ipsilateral amplitudes for HA and LA across memory loads (*M*) *SD* during 600–1,000 ms.

**Memory load**	**HA**			**LA**		
	**Contralateral**	**Ipsilateral**	***p***	**Contralateral**	**Ipsilateral**	***p***
One	0.49 (0.21)	−0.07 (0.20)	< 0.001	−0.42 (0.21)	−0.60 (0.18)	0.23
Two	0.25 (0.20)	−0.39 (0.18)	< 0.001	−1.01 (0.20)	−1.27 (0.17)	0.08
Three	0.31 (0.21)	−0.51 (0.24)	< 0.001	−1.27 (0.22)	−1.84 (0.17)	< 0.001
Four	0.19 (0.21)	−0.58 (0.22)	< 0.001	−1.46 (0.21)	−1.80 (0.04)	0.07
Five	0.11 (0.23)	−0.53 (0.19)	< 0.001	−1.70 (0.23)	−1.72 (0.2)	0.94

## 4. Discussion

In our study, we used an ERP analysis to investigate differences in visual-spatial working memory caused by changes in the HA of the environment, specifically in immigrants who had been living in HA regions for 3 years after emigrating from lower altitude regions. At the behavioral level, these HA immigrants did not show any drawbacks in accuracy and *K*-score. In ERPs, the CDA amplitude of both groups reached its maximum asymptote when the memory load was four, which suggests both groups reached their maximum VWM capacity when the memory load was four. However, within the time window of 300–450 ms, we observed that the amplitude of contralateral waveforms was larger in the LA group than in the HA group when the memory load was one, three, and four, and there was no such pattern when the memory load was two and five. These results suggest LA individuals may process task-relevant information deeper than HA immigrants. Furthermore, we also found that the amplitude of ipsilateral waveforms was significantly more negative than contralateral waveforms in HA immigrants under each memory load during 600–1,000 ms. These findings provide further evidence that long-term HA exposure may impair the ability to process task-relevant information.

It is common knowledge that the most notable characteristic of VWM is its limitation in capacity, which allows for the retention of only ~3–4 visual stimuli within a brief period (29–31). Interestingly, there were no behavioral impairments detected in HA immigrants, which is consistent with the findings of previous fMRI studies demonstrating that these individuals display structural brain impairment but no behavioral deficits. The authors believed that individuals have a certain compensation mechanism for hypoxia, which involves mobilizing additional attentional resources when performing relevant tasks (5, 6, 32, 33). However, we largely attributed this to the nature of the task itself. Specifically, colorful square images were used as stimuli in this study, while the colors of these squares were randomized, and the underlying rectangular shape remained constant throughout the experiment.

Furthermore, each square was randomly presented on the screen with a constrained distance of 2° between squares within a hemifield. This manipulation required the participants to encode the stimuli of the to-be-remembered side at a weak object-object level, simultaneously binding the colors of the colorful squares with their spatial locations. A previous study observed that the behavioral measures of *K*-score and CDA components in ERPs exhibited distinct characteristics in reflecting VWM capacity during the lateralized change detection task. *K*-scores were more sensitive to changes in the number of features in VWM, while the amplitude of CDA was more sensitive to changes in the number of objects in VWM. Specifically, *K*-scores significantly decreased when the number of features increased from one to two, while CDA amplitude was significantly more negative when they memorized three objects compared to one object (34). These findings are consistent with the observation that the amplitude of CDA increased significantly when the memory load increased from load three to load four in both the HA and LA groups within the time window of 300–450 in our study.

In our study, the CDA amplitude of both groups reached an asymptote at a memory load of four. This result seems to indicate that long-term exposure to HA did not cause any impairment of the VWM capacity of HA immigrants. However, based on the analysis of contralateral and ipsilateral activities, we found that hypoxia affected the VWM of HA immigrants, but this effect was only observed in the contralateral and ipsilateral activities (Table 4). The contralateral activity and the ipsilateral activity represent different markers, with the contralateral activity reflecting the process of task-relevant information and the ipsilateral activity representing the process and suppression of task-irrelevant information. Several studies and a meta-analysis have demonstrated that the ability to filter out task-irrelevant information is important in determining the individual VWM capacity (15, 19, 22).

**Table 4:** Contralateral and ipsilateral amplitudes for HA and LA across memory loads (*M*) *SD* during 300–1,200 ms.

**Memory load**	**Contralateral waveforms**			**Ipsilateral waveforms**		
	**HA**	**LA**	***p***	**HA**	**IA**	***p***
One	0.13 (0.17)	−0.54 (0.17)	0.007	−0.34 (0.17)	−0.674 (0.18)	0.12
Two	−0.11 (0.17)	−1.01 (0.17)	< 0.001	−0.69 (0.17)	−1.27 (0.17)	0.02
Three	−0.09 (0.19)	−1.30 (0.24)	< 0.001	−0.72 (0.17)	−1.40 (0.17)	0.007
Four	−0.17 (0.19)	−1.35 (0.19)	< 0.001	−0.69 (0.17)	−1.26 (0.17)	0.03
Five	−0.29 (0.20)	−1.45 (0.21)	< 0.001	−0.701 (0.18)	−1.25 (0.18)	0.04

Figure 2 shows that LA individuals' contralateral and ipsilateral waveforms are significantly larger than those of HA immigrants for each memory load between 300 and 1,200 ms. It may indicate that the processing degree of task-relevant and task-irrelevant information in HA immigrants may be lower than that in LA individuals. Moreover, HA immigrants showed reduced attentional reactions in visual search tasks (24). We observed a load-dependent effect of CDA amplitude during the time window of 300–450 ms. The analysis of contralateral and ipsilateral activity within this period found that contralateral activity was more evident in the LA group than in the HA group when memory load was one, three, and four, but not when it was two or five. This suggests that LA individuals process task-relevant items more deeply than HA.

Interestingly, we also observed, as shown in Figure 2, that the amplitude of ipsilateral waveforms was significantly larger than that of contralateral waveforms in each memory load for HA immigrants within the time window of 600–1,000 ms, but we made no such observation for LA individuals. This provides strong evidence that long-term HA exposure affects the representation of task-relevant information. In a study on patients with Parkinson's disease, it was observed that their ability to filter distractions during tasks was reduced. Specifically, the patients showed similar CDA amplitude and *K*-scores at a memory load of two (two red items) and four (two red and two green items) (35). This finding suggests that patients with Parkinson's disease have difficulty suppressing irrelevant information in VWM tasks. HA immigrants may have a similar impairment in filtering out irrelevant information from the display arrays and a reduced ability to allocate attention. Vogel et al. found that low-capacity individuals exhibited almost equal amplitude of CDA between the condition that included four task-relevant items and the typical lateralized change detection condition where two task-relevant items and two distractors were simultaneously presented (19), which indicates that individuals with low VWM capacity have difficulties filtering out task-irrelevant information. This explains the equal CDA amplitude observed between the HA and LA groups, while the more negative amplitude of ipsilateral waveforms for HA immigrants suggests that the effect of task-irrelevant items may have even suppressed the effect of task-relevant items. The executive attention theory of working memory (14, 36, 37) postulates that cognitive control is key to maintaining more items in VWM, as it is required for actively maintaining task goals. However, long-term HA exposure can impair cognitive control (11, 17), leading to insufficient attention being allocated to task-relevant and task-irrelevant information. In summary, the findings suggest that long-term HA exposure can lead to a reduction in the representation of task-relevant information in HA immigrants. This reduction is likely due to a decreased ability to filter out task-irrelevant information and impaired cognitive control, which are both key factors for maintaining more items in VWM.

A potential explanation for why HA individuals showed a reduced capacity to store task-relevant information is that prolonged exposure to HA may require adaptation, which could consume the limited resources needed for storing information necessary for tasks with high memory loads and numerous distractors. Hypoxia has been shown to impair cognitive function (11, 16, 25, 38), particularly attention (24, 25, 39). As an advanced cognitive function, working memory is also known to impact attention. Previous studies conducted by our lab have found that long-term exposure to HA results in a reduction in the ability to allocate attentional resources in competitive situations (39). Furthermore, hypoxia has been shown to impair individuals' ability to perceive stimuli, suggesting that long-term HA exposure impairs spatial attention ability (25).

Additionally, hypoxia also affects the allocation of attentional resources to different stimuli and conflict resolution, which are critical for WM. When individuals are exposed to hypoxia, they may experience a reduction in their ability to attend to stimuli and allocate attentional resources to different stimuli, leading to impairments in their working memory. The decreased ability to resolve conflicts (11, 34) further impairs WM by selecting relevant information and suppressing irrelevant information.

The capacity of WM changes over time, and individuals can improve their performance on WM tasks by adopting various approaches to reinforce their memory (40–42), such as practicing the tasks to familiarize themselves with the configuration of the task's stimuli, including the relationship between objects (31). Therefore, future studies need to consider these factors and establish training programs to explore ways to alleviate the effects of hypoxia on WM.

## 5. Conclusion

In conclusion, the present study conducted an ERP analysis to investigate the performance of VWM in immigrants who had lived in a HA environment for the past 3 years. These findings revealed that the attentional resources of individuals exposed to long-term HA are captured more by task-irrelevant information, potentially due to impaired inhibitory control, which makes it difficult for them to exclude the interference of task-irrelevant information.

## Data availability statement

The raw data supporting the conclusions of this article will be made available by the authors, without undue reservation.

## Ethics statement

The studies involving human participants were reviewed and approved by the Research Ethics Committee in the Academy of Tibet University. The patients/participants provided their written informed consent to participate in this study.

## Author contributions

XB and HM: conceptualization. XB and XL: software and data collection. XB: formal analysis and writing—original draft. DZ and ML: writing—editing. All authors contributed to the article, read and approved the submitted version.
